# Perceived COVID-19 crisis strength and teachers’ emotional labor: mediating role of interpersonal stress and moderating role of gender

**DOI:** 10.3389/fpsyg.2024.1425606

**Published:** 2024-11-01

**Authors:** Yantao Shi, Qingle Hu, Qinghuan Tao

**Affiliations:** ^1^College of Innovation and Entrepreneurship, Guangxi Minzu University, Nanning, China; ^2^School of Rail Transit Engineering, Zhengzhou Technical College, Zhengzhou, China; ^3^School of Education Science, Guangxi Minzu University, Nanning, China

**Keywords:** perceived COVID-19 crisis strength, emotional labor, interpersonal stress, gender, primary and secondary school teachers

## Abstract

Perceived COVID-19 crisis strength has been associated with teachers’ emotional labor, but little is known about the mediating and moderating mechanisms underlying this association. This study aimed to explore whether interpersonal stress would mediate the relationship between perceived COVID-19 crisis strength and emotional labor, and whether gender would moderate the indirect pathway between perceived COVID-19 crisis strength and interpersonal stress. Participants were 889 primary-and secondary-school teachers from Guangxi, China, selected using convenient sampling method. They completed measurements regarding emotional labor, interpersonal stress, and perceived COVID-19 crisis strength. Results showed that perceived COVID-19 crisis strength was negatively associated with deep acting but not surface acting, and this association was fully mediated by interpersonal stress. Moreover, the indirect relationship between perceived COVID-19 crisis strength and interpersonal stress was moderated by gender, with the indirect relationship being stronger for male teachers than for female teachers. This study illuminates the psychological mechanisms underlying the association between perceived COVID-19 crisis strength and emotional labor, enriching our understanding of this association and gender differences among primary and secondary school teachers.

## 1 Introduction

In the workplace, employees interacting with clients are typically required to perform emotional labor by expressing socially or organizationally desired emotions and regulating their emotional stress associated with work. Emotional labor is defined as “the management of feeling to create a publicly observable facial and bodily display” (Hochschild, 1983, p. 7). Deep acting and surface acting are two common strategies of emotional labor performed by employees to manage their emotions (Hochschild, 1983; Grandey, 2003). Deep acting is regarded as an adaptive emotion regulation strategy by which individuals authentically modify their internal feelings to align with the desired emotional expression (Hülsheger and Schewe, 2011; Zheng et al., 2020). On the contrary, surface acting is a maladaptive strategy which involves suppressing genuine emotions and instead displaying false or insincere expressions in response to organizational expectations or social norms (Hülsheger and Schewe, 2011; Humphrey et al., 2015; Grandey and Melloy, 2017; Huyghebaert et al., 2018).

Teaching is an emotionally demanding profession that requires teachers to perform high levels of labor emotion when interacting with students (Wang et al., 2021; Berheide et al., 2022; Shi and Ouyang, 2024). Previous studies have revealed that the significant role of emotional labor in teacher well-being (e.g., job satisfaction) (Huang et al., 2019; Yin et al., 2019; Guy, 2020; Wang et al., 2021), self-efficacy (Lee and van Vlack, 2018; Shi and Ouyang, 2024), and teaching effectiveness (Wang et al., 2023), highlighting that deep acting is less harmful and more beneficial than surface acting. Recent studies have shown that the COVID-19 pandemic as an antecedent impacts teachers’ emotional labor (Berheide et al., 2022). Researchers introduced the concept of perceived COVID-19 crisis strength, suggesting that the impact of COVID-19 pandemic on individuals’ physical and mental health depends on individuals’ perception and evaluation of the pandemic (Liu et al., 2021).

The perceived COVID-19 crisis strength affects the two strategies of emotional labor differently. According to the cognitive-affective system and conservation of resources theory, when the perceived COVID-19 crisis strength is low, individuals tend to adopt deep acting (Germani et al., 2020). This alignment aids in the efficient conservation of resources, enabling individuals to better meet external emotional demands and reduce emotional exhaustion (Hobfoll, 1989; Goldberg and Grandey, 2007). In contrast, when the perceived COVID-19 crisis strength is high, individuals exhibit anxious emotional states and tend to resort to ostensible behavior. In this scenario, the dissonance between internal feelings and external emotional expressions diminishes the sense of self-authenticity, leading to emotional dysregulation and exhaustion (Zapf, 2002). Empirical research supports the different impacts of perceived COVID-19 crisis strength on emotional labor. Lower perceived COVID-19 crisis strength is associated with the more use of deep acting (Chiu et al., 2021), while higher perceived COVID-19 crisis strength is associated with surface acting (González-Castro et al., 2021). This indicates that teachers with intense pandemic perception strength, experiencing negative emotions, may lead to employee emotional exhaustion and burnout (Ashforth and Humphrey, 1993).

The COVID-19 pandemic has intensified emotional labor demands for teachers, presenting challenges in teaching and experiencing burnout (Robinson et al., 2022). Previous meta-analyses have shown a significant positive correlation between teachers’ perceived risk during the pandemic and their anxiety (Yu et al., 2021), while a significant negative impact has been observed between teachers’ pandemic perception and positive emotional regulation (Messineo and Tosto, 2022). Empirical studies have demonstrated that COVID-19 pandemic affects the use of emotional labor. For instance, under the stress and anxiety of the pandemic, teachers are more likely to adopt surface acting than deep acting, which results in increased burnout and decreased job satisfaction (Daumiller et al., 2021; Güler et al., 2023). Research indicates that teachers’ perceived strength of the COVID-19 pandemic directly affects their emotional labor strategies (Du et al., 2023), with individuals perceiving higher crisis strength experiencing greater anxiety and emotional tension (Fu et al., 2021). These studies suggest that perceived COVID-19 crisis strength may be a significant predictor of emotional labor. However, little empirical research has explored the mechanisms underlying the relationship between perceived COVID-19 crisis strength and emotional labor among teachers. Thus, we used a sample of teachers to examine whether interpersonal stress can mediate this relationship and whether gender can moderate the indirect relationship between perceived COVID-19 crisis strength and interpersonal stress.

### 1.1 The mediating effect of interpersonal stress

Interpersonal stress refers to the stress that teachers perceive from interpersonal relationships in the workplace (Christina et al., 2001). The cognitive interaction theory posits that interpersonal stress is influenced by how individuals perceive their surrounding environment and by the manner in which these perceptions impact their emotions and behaviors (Jennifer et al., 2013). According to this theory, we propose that perceived COVID-19 crisis strength would be positively associated with teachers’ interpersonal stress. During the COVID-19 pandemic, teachers who perceive the pandemic as a serious threat may experience higher levels of emotional stress and anxiety (Ozamiz-Etxebarria et al., 2021; Pressley et al., 2021; Vargas Rubilar and Oros, 2021). In addition, the COVID-19 pandemic has led schools to shift to online or hybrid teaching models, requiring rapid adaptation to new technologies and teaching methods. This transition not only increases workload but also poses great challenges to the interaction between teachers and students, thereby increasing interpersonal stress (Vargas Rubilar and Oros, 2021; Yang, 2021).

Interpersonal stress may be significantly associated with teachers’ emotional labor. Teachers who face interpersonal stress may need to engage in less deep acting and more surface acting to adapt to workplace demands. According to the person-environment fit theory, individuals’ job performance depends on the match between their abilities and environmental demands (Greguras and Diefendorff, 2009). During the pandemic, teachers often experience a mismatch between their capabilities, skills, and emotional resources, and the rapidly changing work environment. They may perceive the new demands of the teaching environment, including coping with pressure from colleagues and administrative management. This may lead to a greater necessity for surface acting to maintain a professional image in the online teaching environment, thereby conserving teachers’ emotional resources. Moreover, prolonged stress and uncertainty could deplete teachers’ emotional resources (Chang, 2009), thereby diminishing their capacity for deep acting (Chang, 2009; Huang et al., 2015). In a context of limited resources, teachers may prioritize surface acting that consumes fewer resources (Lee, 2019). Furthermore, a meta-analysis has revealed the positive correlation between interpersonal stress and surface acting (Codou et al., 2016), and the negative correlation with deep acting (David et al., 2007).

Based on hypotheses that perceived COVID-19 crisis strength would be significantly correlated with interpersonal stress, and that interpersonal stress would be significantly correlated with teachers’ emotional labor, we propose that teachers’ interpersonal stress would play a mediating role in the relationship between perceived COVID-19 crisis strength and teachers’ emotional labor.

### 1.2 The moderating effect of gender

Although significant relationship between COVID-19 pandemic and stress has been consistently identified in previous studies on teachers (Ozamiz-Etxebarria et al., 2021; Ma et al., 2022; Robinson et al., 2022), none of the studies have explored the moderating role of gender in the relationship between perceived COVID-19 crisis strength and teachers’ interpersonal stress in the workplace. As social cognitive theory posits that individuals’ behaviors (e.g., interpersonal stress) are influenced by the interaction of environmental (e.g., perceived COVID-19 crisis strength) and personal factors (e.g., gender) (Bandura, 2001). According to this theory, we proposed that gender may moderate the relationship between perceived COVID-19 crisis strength and teachers’ interpersonal stress, with the relationship being much stronger for male teachers than for female teachers. There are several reasons for this speculation. First, previous studies emphasized the role of gender differences in both COVID-19 pandemic-related stress (Alsharawy et al., 2020; Klapproth et al., 2020; Rinfret et al., 2022) and coping strategies used (Graves et al., 2021). Ample studies have shown that, compared to male teachers, female teachers experienced greater COVID-19 related stress (Klapproth et al., 2020; Stachteas and Stachteas, 2020; Oducado et al., 2021; Tharp et al., 2021). However, they tend to employ adaptive coping strategies, such as seeking social support, to copy with the stress, whereas men tend to resort to maladaptive coping strategies like denial in managing stressful situations (Dakhli et al., 2013; Alhija, 2015; Ferguson et al., 2017; Klapproth et al., 2020). Therefore, male teachers may report higher levels of interpersonal stress than female teachers in facing the COVID-19 epidemic.

### 1.3 Purpose of this study

**Figure 1:**
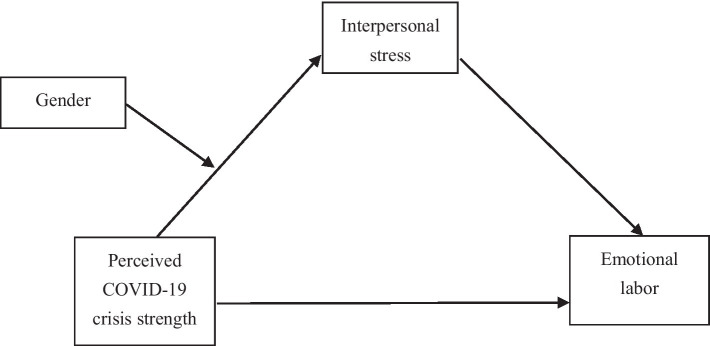
The assumptive moderated mediation model.

In summary, this study investigates the mediating role of interpersonal stress in the relationship between perceived COVID-19 crisis strength and teachers’ emotional labor, and the moderating role of gender in the indirect relationship between perceived COVID-19 crisis strength and teachers’ interpersonal stress (see Figure 1). This study is helpful to answer the psychological mechanisms underlying the relationship between perceived COVID-19 crisis strength and teachers’ emotional labor.

## 2 Methods

### 2.1 Participants

This study applied the convenience sampling method to recruit teachers from primary and secondary schools in Guangxi in China. Eight hundred and eighty-nine teachers, aged 22—55 years old (*M* = 40.21, *SD* = 10.24), were recruited from 48 primary and secondary schools. Among them, 65.6% were females and 34.4% were males. Concerning their educational levels, 239 (26.9%) had completed senior high school level and 650 (72.1%) had completed a bachelor’s degree or higher level. In terms of their teaching experience, 303 (34%) had taught for less than 10 years, 181 (20%) had taught for 10 to 20 years, and 405 (46%) had taught for more 20 years.

### 2.2 Measures

#### 2.2.1 Emotional labor

The Emotional Labor Strategies Scale (ELS) was employed to assess teachers’ emotional labor (Crandey, 2003). The scale has 11 items including two dimensions: deep acting (6 items, e.g., “The emotion that I show to my students is my true feeling.”) and surface acting (5 items, e.g., “When facing my students, I often need to disguise my emotions.”). Items are rated on a 5-point Likert scale, ranging from1 (Never) to 5 (Always). The second-order CFA model showed that the ELS had adequate construct validity, with *χ^2^*/*df* = 2.11, RMSEA = 0.038, CFI = 0.87, GFI = 0.88, IFI = 0.87, AGFI = 0.85. In this study, Cronbach’s alpha for subscales of the deep acting and the surface acting were 0.88 and 0.89, respectively.

#### 2.2.2 Perceived COVID-19 crisis strength

The Perceived COVID-19 Crisis Strength Scale (PCCS) was utilized to assess teachers’ perceived COVID-19 crisis strength (Liu et al., 2021). The scale comprises 11 items including three subscales: novelty (4 items, e.g., “There is a series of understandable steps to deal with the COVID-19 pandemic crisis.”), disruption (4 items, e.g., “The COVID-19 pandemic crisis has made me pause and reflect on how to respond.”), and criticality (4 items, e.g., “The COVID-19 pandemic crisis is a matter that I need to prioritize.”). Each item is rated on a 5-point Likert scale, ranging from 1 (Strongly disagree) to 5 (Strongly agree). Higher average scores indicate higher levels of perceived COVID-19 crisis strength. The second-order CFA model showed that the PCCS had good construct validity, with *χ^2^*/*df* = 4.13, RMSEA = 0.06, CFI = 0.91, GFI = 0.92, IFI = 0.91, AGFI = 0.92. In this study, Cronbach’s alpha for subscales of the novelty, disruption, and criticality were 0.88, 0.75, and 0.72.

#### 2.2.3 Interpersonal stress

The interpersonal stress subscale of the Occupational Stress Indicator (OSI) (Williams and Cooper, 1998) was revised to measure teachers’ interpersonal stress. The scale consists of 4 items (e.g., “Most colleagues are not friendly or interested in me.”). Each item was rated on a 7-point Likert scale from 1 (Never) to 7 (Very frequently), with higher average scores indicating higher levels of interpersonal stress. The second-order CFA model showed that the OSI had adequate construct validity, with *χ^2^*/*df* = 1.59, RMSEA = 0.03, CFI=,88, GFI = 0.86, IFI = 0.85, AGFI = 0.99. In this study, Cronbach’s alpha was 0.96.

### 2.3 Procedure

This study was approved by the research Ethics Committee of the authors’ university. Written informed consent was obtained from all participants before data collection. Participants completed questionnaires regarding the perceived COVID-19 crisis strength, emotional labor, and interpersonal stress through a commonly used online survey platform^1^ in July 2022. They were informed of the survey’ s anonymity and confidentiality, with the freedom to discontinue participation at any time.

### 2.4 Statistical analyses

First, descriptive statistics and Pearson correlation among the study variables were analyzed. Second, the mediating effect of interpersonal stress in the relationship between perceived COVID-19 crisis strength and emotional labor was examined using the PROCESS macro (Model 4) (Hayes, 2013). Third, the moderating effect of gender on the indirect relationship between perceived COVID-19 crisis strength and interpersonal stress was analyzed using the PROCESS macro (Model 7) (Hayes, 2013). The bootstrapping method was used to examine the significance of the effects in Model 4 and Model 7 based on 5,000 resamples. A effect is significant if the 95% confidence intervals (95%*CI*s) do not include zero. All analyses were conducted using SPSS 17.0.

## 3 Results

### 3.1 Descriptive statistics and correlational analyses

The descriptive statistics were presented in Tables 1, 2, separately for gender and educational level, respectively. To examine whether strategies of emotional labor differed by gender, an independent sample *t*-test was conducted and the results showed no significant differences in deep acting (*t* = 1.69, *p* = 0.09) and surface acting (*t* = −1.56, *p* = 0.12) between gender. Besides, an ANOVA was conducted to determine whether strategies of emotional labor differed by educational level. The results showed that teachers with more than 20 years of teaching experience performed greater use of deep acting than those with less than 10 years of teaching experience. Additionally, teachers with more than 10 years of teaching experience performed greater use of surface acting than those with less than 10 years of teaching experience.

**Table 1:** Teaching experience difference in emotional labor.

Variables	Teaching experience	*M*	*SD*	*F*	*p*	*Post hoc* comparison
Deep acting	1. Less than 10 years	3.27	0.84	4.47	0.012	1 < 3
	2. 10–20 years	3.58	0.67			
	3. More than 20 years	3.61	0.74			
Surface acting	1. Less than 10 years	2.40	0.78	18.49	<0.001	1 < 3, 1 < 2
	2. 10–20 years	2.53	0.76			
	3. More than 20 years	2.58	0.86			

*p < 0.05.*

**Table 2:** Gender difference in emotional labor.

Variables	Gender	*M*	*SD*	*t*	*p*
Deep acting	Male	3.44	0.67	1.69	0.09
	Female	3.53	0.67		
Surface acting	Male	2.58	0.88	−1.56	0.12
	Female	2.49	0.79		

*p < 0.05.*

The correlation analysis reveals that perceived COVID-19 crisis strength was negatively associated with teachers’ deep acting (*r* = −0.11, *p* < 0.001), but not teachers’ surface acting (*r* = 0.06, *p* > 0.05). Perceived COVID-19 crisis strength was positively with teachers’ interpersonal stress (*r* = 0.25, *p* < 0.001). Interpersonal stress was negatively associated with teachers’ deep acting (*r* = −0.23, *p* < 0.001) but positively associated with teachers’ surface acting (*r* = 0.17, *p* < 0.001). Gender was negatively associated with perceived COVID-19 crisis strength (*r* = −0.08, *p* < 0.05) and interpersonal stress (*r* = −0.13, *p* < 0.001) (Table 3).

**Table 3:** Correlational analyses for the study variables.

Variables	*M*	*SD*	1	2	3	4	5
1. Gender	—	—	1				
2. Surface acting	2.52	0.82	−0.06	1			
3. Deep acting	3.50	0.78	0.06	0.28^***^	1		
4. Interpersonal stress	2.48	1.14	−0.13^***^	0.17^***^	−0.23^***^	1	
5. Perceived COVID-19 crisis strength	2.85	0.41	−0.08^*^	0.06	−0.11^**^	0.25^***^	1

*p < 0.05, **p < 0.01, ***p < 0.001.

### 3.2 Testing for mediation effect

As Table 4 showed, perceived COVID-19 crisis strength was positively associated with interpersonal stress (*β* = 0.25, *t* = 7.80, *p* < 0.001) (see Model 2 of Table 4), which was negatively associated with deep acting (*β* = −0.22, *t* = −6.53, *p* < 0.001) (see Model 3 of Table 4). The direct relationship between perceived COVID-19 crisis strength and deep acting became non-significant (*β* = −0.06, *t* = −1.65, *p* = 0.10). The bias-corrected percentile bootstrap method also showed that the indirect effect of perceived COVID-19 crisis strength on deep acting via interpersonal stress was significant (*ab* = −0.06, SE =0.02, 95%*CI*s = [−0.08, −0.03]), but the direct effect of perceived COVID-19 crisis strength on deep acting was not significant (*ab* = −0.05, *SE* = 0.03, 95%*CIs* = [−0.12, 0.01]). Therefore, the results above indicated that interpersonal stress fully mediated the relationship between perceived COVID-19 crisis strength and deep acting.

**Table 4:** Testing the mediation effect of interpersonal stress.

Variables	Model 1 (deep acting)				Model 2 (interpersonal stress)				Model 3 (deep acting)			
	*β*	SE	LLCI	ULCI	*β*	SE	LLCI	ULCI	*β*	SE	LLCI	ULCI
Perceived COVID-19 crisis strength	−0.11	0.03	−0.18	−0.04	0.25	0.033	0.19	0.32	−0.06	0.03	−0.12	0.01
Interpersonal stress									−0.22	0.03	−0.29	−0.15
*R^2^*	0.01				0.06				0.06			
*F*	11.13^***^				60.9^***^				27.12^***^			

β are standardized coefficients. SE, standard error; LLCI, lower limit of the 95% confidence interval; ULCI, upper limit of the 95% confidence interval. ***p < 0.001.

### 3.3 Testing for moderation effect

**Figure 2:**
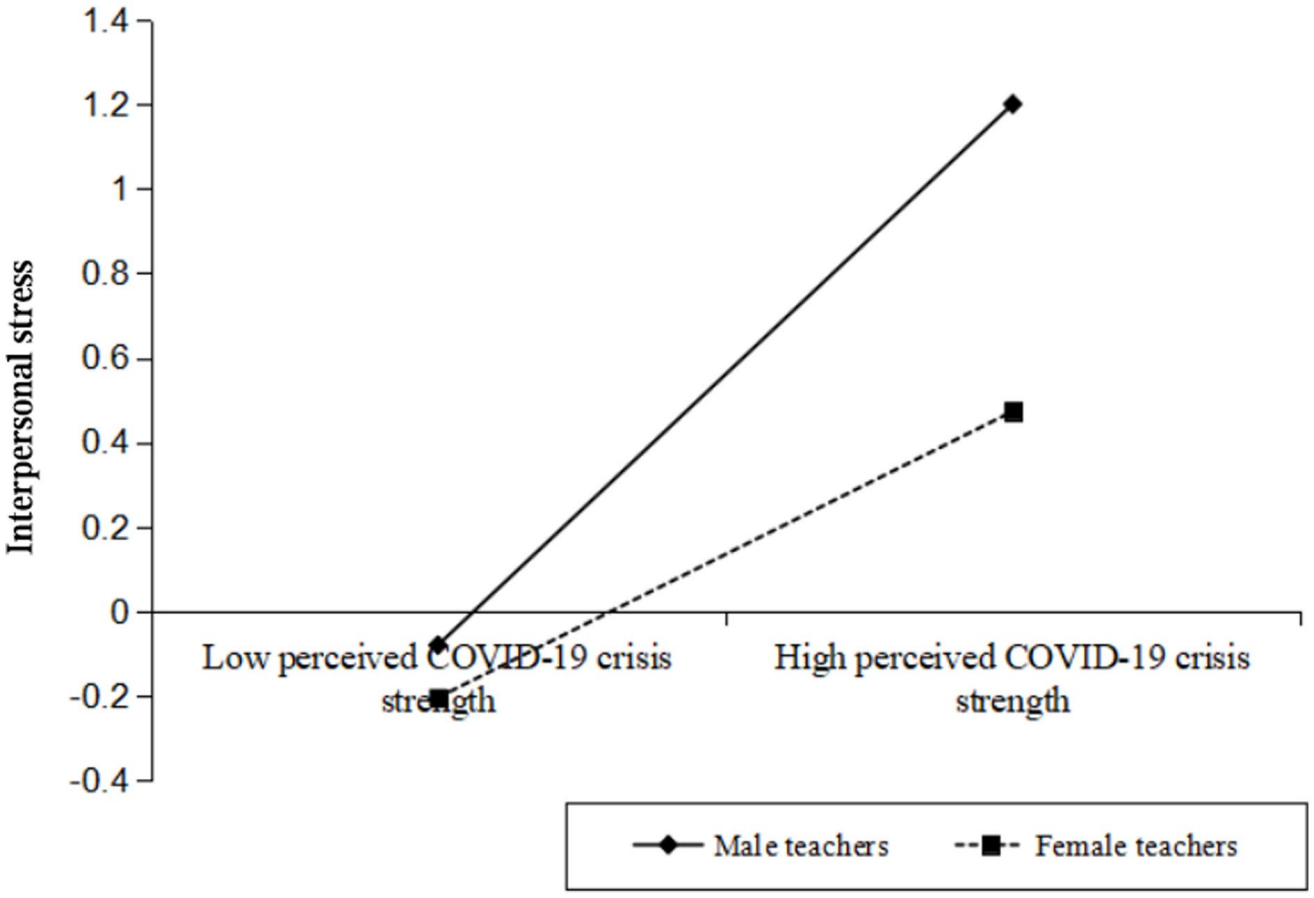
Gender moderates the indirect relationship between perceived COVID-19 crisis strength and interpersonal stress.

As shown in Table 5, perceived COVID-19 crisis strength was positively associated with interpersonal stress, *β* = 0.49, *t* = 4.35, *p* < 0.001, and more importantly, the interaction effect between perceived COVID-19 crisis strength and gender was significant, *β* = −0.15, *t* = −2.27, *p* < 0.05. Thus, the indirect effect of perceived COVID-19 crisis strength on interpersonal stress was moderated by gender. For descriptive purposes, this study plotted perceived COVID-19 crisis strength on interpersonal stress, separately at male teachers and female teachers (Figure 2). Simple slope tests showed the effect of perceived COVID-19 crisis strength on interpersonal stress was stronger for male teachers (*β*simple = 0.64, *t* = 3.61, *p* < 0.001) than for female teachers (*β*simple = 0.34, *t* = 6.47, *p* < 0.001).

**Table 5:** Testing the moderated mediation effect of perceived COVID-19 crisis strength on interpersonal stress.

Variables	Model (interpersonal stress)			
	*β*	SE	LLCI	ULCI
Perceived COVID-19 crisis strength	0.49	0.11	0.27	0.71
Gender	−0.21	0.07	−0.35	−0.08
Perceived COVID-19 crisis strength × gender	−0.15	0.07	−0.28	−0.02
*R^2^*	0.08			
*F*	25.84^***^			

β are standardized coefficients. SE, standard error; LLCI, lower limit of the 95% confidence interval; ULCI, upper limit of the 95% confidence interval. Each column is a regression model that predicts the criterion at the top of the column. ***p < 0.001.

The bias-corrected bootstrap analysis further revealed that the indirect effect of perceived COVID-19 crisis strength on interpersonal stress was moderated by gender. For male teachers, the indirect relationship between perceived COVID-19 crisis strength and interpersonal stress was stronger, *β* = −0.07, *SE* = 0.02, 95%*CI*s = [−0.11, −0.04], while for female teachers, this indirect relationship was relatively weaker, *β* = −0.04, *SE* = 0.01, 95%*CI*s = [−0.07, −0.02].

## 4 Discussion

Although empirical studies have revealed the significant impact of perceived COVID-19 crisis strength on teachers’ emotional labor (María and Ana, 2020), limited research has been conducted on the psychological mechanisms underlying this effect. This study investigated the mediating role of interpersonal stress in the relationship between COVID-19 perception strength and teachers’ emotional labor, as well as the moderating role of gender in the indirect relationship between perceived COVID-19 crisis strength and interpersonal stress. The results revealed a negative correlation between perceived COVID-19 crisis strength and deep acting, but not with surface acting. Additionally, interpersonal stress fully mediated the relationship between perceived COVID-19 crisis strength and teachers’ deep acting. Furthermore, gender significantly moderated the indirect relationship between perceived COVID-19 crisis strength and interpersonal stress. Specifically, this indirect relationship was stronger for male teachers than for female teachers. We will discuss each of these findings in the following section.

### 4.1 The mediating effect of interpersonal stress

This study found a significant positive correlation between perceived COVID-19 crisis strength and interpersonal stress, which was negatively correlated with deep acting. In other words, interpersonal stress played a mediating role in the relationship between perceived COVID-19 crisis strength and deep acting. The findings indicated that teachers with high levels of perceived COVID-19 crisis strength are more likely to experience high levels of interpersonal stress, subsequently leading them to engage in less deep acting. Therefore, interpersonal stress may serve as one of the explanatory mechanisms for why teachers exhibit less deep acting when they perceive higher levels of COVID-19 crisis strength. Previous empirical studies have not explored the underlying mechanisms of this linkage.

Besides the overall mediating effect of interpersonal stress, it is important to note the two separate indirect paths of the mediating process. Firstly, this study revealed a significant positive correlation between perceived COVID-19 crisis strength and teachers’ interpersonal stress. Amidst uncertainty and rapid changes in teaching methods and work patterns, along with barriers to emotional exchanges among teachers, leaders, and students, heightened perception of the COVID-19 pandemic exacerbates interpersonal stress for teachers. During the COVID-19 pandemic, teachers face potential concerns regarding the health and safety of themselves, their families and their students. This is especially true for teachers who are on high alert for a pandemic. They experience heightened anxiety and are uneasy about the increased risk of infection in a school environment, which can lead to increased interpersonal stress. In the context of a pandemic, collaboration and support among teachers becomes particularly critical. However, teachers who were highly concerned about the pandemic may have relied more on their colleagues for assistance, potentially increasing their interpersonal stress. Moreover, during the COVID-19 pandemic, the teacher-parent relationship shifted from “unilateral cooperation” to “mutual benefit,” leading to increased interpersonal stress (María and Ana, 2020). Consequently, teachers with high perceived COVID-19 crisis strength are more likely to report elevated interpersonal stress (Robinson et al., 2022), consistent with existing research (Santamaria et al., 2021). Additionally, the cognitive interaction theory of stress could explain this finding, suggesting that interpersonal stress is influenced by cognitive evaluations (Lazarus and Folkman, 1984). Teachers perceiving higher COVID-19 crisis strength undergo heightened cognitive evaluations of its uncertainty, destructiveness, and novelty, leading to increased anxiety, emotional tension, and interpersonal stress.

Secondly, this study revealed a significant negative correlation between interpersonal stress and teachers’ deep acting, indicating that teachers with higher levels of interpersonal stress tend to engage in less deep acting. The reason could be that high levels of interpersonal stress deplete teachers’ emotional resources, hindering their ability to employ adaptive emotion regulation strategies such as deep acting. According to the conservation of resources theory (Schunk, 1989), individuals tend to conserve their resources in situations of scarcity. As a result, teachers experiencing high levels of interpersonal stress may opt for resource-conserving coping strategies, such as surface-based rather than deep-based. Previous studies also indicate that teachers facing high interpersonal stress are more likely to engage in surface acting, such as hiding or disguising their true emotions, rather than employing deep emotional regulation to cope with emotional challenges (Grandey et al., 2004; Philipp and Schüpbach, 2010). Teachers often face significant workloads, which can lead to emotional anxiety and fatigue. This emotional burden can be further exacerbated when interpersonal stress increases. Due to time and energy constraints, teachers may find it challenging to effectively engage in deep acting. Especially when dealing with highly stressful relationships, they may struggle to effectively manage negative emotions and stress, and thus tend to adopt short-term, superficial emotional regulation strategies rather than engage in deep emotional regulation. The nature of the teaching profession requires the maintenance of professionalism and stability in various interpersonal interactions. When dealing with student issues or communicating with parents, teachers may need to control their emotions to maintain a positive teaching environment and relationship. Consequently, as interpersonal stress increases, teachers may be more inclined to choose surface-level performance strategies to maintain their job performance and professional image.

### 4.2 The moderating effect of gender

This study found that the indirect relationship between perceived COVID-19 crisis strength and interpersonal stress was moderated by gender. Specifically, this indirect relationship was stronger for male teachers than for females. This may be because male teachers, compared to females, are more concerned not only about COVID-19 infection but also about the economic strain the crisis has placed on family financial stability. These results support the viewpoints of Lewis (2007) and Baldwin (1992). Due to the traditional “strong” role, male teachers may be reluctant to express or share their worries and stress. During the COVID-19 pandemic, male teachers may internalize feelings instead of seeking support or expressing vulnerability, leading to increased interpersonal stress. In contrast, female teachers are more inclined to comprehensively assess pandemic information, share concerns, and recognize its wide-ranging impact on relationships and social interactions. When facing a pandemic crisis, female teachers excel in seeking and utilizing social support networks to alleviate interpersonal stress.

### 4.3 Limitations and future directions

This study has several limitations that should be addressed in future studies. First, this study was a cross-sectional design, limiting the causal inference. Future studies should employ longitudinal or experimental design to make a causal conclusion. Second, this study used self-reported questionnaires to measure teachers’ perceived COVID-19 crisis strength, emotional labor, and interpersonal stress, potentially introducing response bias. Future studies should use a multi-method approach to arrive at more persuasive conclusions. Third, participants in this study are limited to primary and secondary school teachers from Guangxi in China, so our findings might be not generalizable to other populations. Future studies need to examine and improve the generalizability of these findings in teachers from different educational stages and regions. In addition, further research could investigate other potential variables that may mediate or moderate the relationship between perceived COVID-19 crisis strength and emotional labor, such as psychological resilience and social support. This would provide a more comprehensive understanding of the factors that influence emotional labor.

### 4.4 Implications

Despite these limitations, this study has theoretical and practical implications. Theoretically, it explores the relationship between perceived COVID-19 crisis strength and teachers’ emotional labor, extending previous research (David et al., 2007; Zheng et al., 2020) by providing a more comprehensive understanding of the impact of pandemic-related stressors on teachers’ emotion regulation strategies in the workplace. Additionally, we demonstrate the mediating effect of interpersonal stress in this relationship, as well as the moderating effect of gender on the relationship between perceived COVID-19 crisis strength and teachers’ interpersonal stress. These findings contribute to a better understanding of how perceived COVID-19 crisis strength relates to teachers’ emotional labor and the gender differences in its influence on interpersonal stress.

Practically, this study can inform effective approaches to address the impact of perceived COVID-19 crisis strength on teachers’ emotional labor, especially considering gender differences. For example, creating dedicated communication platforms for teachers to share experiences, seek support, and build positive collegial relationships can help address interpersonal stress. Moreover, the study highlights a correlation between perceived COVID-19 crisis strength and increased interpersonal stress among male teachers. Therefore, schools should provide gender-sensitive support and training for male teachers to help them better cope with interpersonal stress, thereby enhancing their capacity for emotional labor.

## 5 Conclusion

We concluded that perceived COVID-19 pandemic crisis strength was associated with reduced use of deep acting but not surface acting among primary and secondary school teachers. Furthermore, this association was fully mediated by interpersonal stress. Importantly, we found that gender moderated the association between perceived COVID-19 pandemic crisis strength and interpersonal stress, with the association being stronger among male teachers compared to females. Our findings contribute to a better understanding of the impact of perceived COVID-19 pandemic crisis on emotional labor and its gender differences among primary and secondary school teachers.

## Data Availability

The original contributions presented in the study are included in the article/supplementary material, further inquiries can be directed to the corresponding author.

## Footnotes

^1^ www.wjx.cn
